# Post‑vaccination Acute Pancreatitis in an Infant: A Report of a Rare Case and Brief Literature Review

**DOI:** 10.7759/cureus.109295

**Published:** 2026-05-20

**Authors:** Ghalib Nashaat El Hunjul, Poorvi Gupta, Khurshid Khan

**Affiliations:** 1 General Pediatrics, Al Qassimi Women’s and Children’s Hospital, Sharjah, ARE; 2 Pediatric Gastroenterology, Al Qassimi Women’s and Children’s Hospital, Sharjah, ARE

**Keywords:** acute pancreatitis, mmr vaccine, pediatric pancreatitis, post‑vaccination pancreatitis, vaccine adverse event

## Abstract

Acute pancreatitis (AP) is increasingly recognized in the pediatric population, although it remains uncommon in infants and young children. In this age group, diagnosis is often delayed because clinical manifestations, such as irritability, vomiting, and feeding intolerance, are nonspecific and may occur in the absence of classic abdominal pain. Most pediatric cases are mild and self‑limiting; however, severity can range from mild to severe depending on the presence and duration of organ dysfunction and the development of local or systemic complications at presentation.

A wide spectrum of etiologies has been described in pediatric AP, acute recurrent pancreatitis (ARP), and chronic pancreatitis (CP). However, vaccination is among the least frequently reported associations, with only isolated case reports in the literature. Establishing causality in such instances is challenging, and a thorough evaluation for more common etiologies remains essential.

We report the case of a 12‑month‑old girl who developed AP three days after receiving the measles, mumps, and rubella (MMR) vaccine, which highlights the importance of maintaining a broad differential diagnosis.

## Introduction

Acute pancreatitis (AP) is increasingly recognized in pediatrics, with multiple series describing a rising incidence over the last two decades; nevertheless, AP remains uncommon in infants and young toddlers, where clinical recognition is challenging because symptoms are often nonspecific (irritability/inconsolable crying, vomiting, feeding refusal) rather than classic adult-type epigastric pain [1,2]. Diagnosis in children is commonly based on the International Study Group of Pediatric Pancreatitis: In Search for a Cure (INSPPIRE) definition requiring at least two of three criteria: (i) abdominal pain compatible with AP (or pain-equivalent behavior in preverbal children), (ii) serum amylase and/or lipase ≥3× upper limit of normal (ULN), and (iii) imaging findings consistent with AP [1].

Most pediatric cases are mild, but severity is classified into mild, moderately severe, or severe. Mild AP is the most common form and is characterized by the absence of organ failure or local/systemic complications, typically resolving within the first week of illness. Moderately severe AP involves either transient organ dysfunction lasting no more than 48 hours or the development of local complications (such as pancreatic fluid collections or necrosis) or systemic complications, including worsening of preexisting conditions like lung or kidney disease. Severe AP is defined by persistent organ failure lasting longer than 48 hours, which may affect one or multiple organs and can develop later in the course of the disease [3]. However, over the past 20 years, reports from several regional centers have indicated that the incidence of pediatric AP is 3-13 cases per 100,000 individuals, overlapping with the lower limit of the adult incidence rate (5-60 cases per 100,000 individuals) [4].

Management is largely supportive (fluids, analgesia, monitoring), and contemporary pediatric guidance encourages early enteral/oral feeding as tolerated rather than prolonged fasting, while avoiding prophylactic antibiotics in uncomplicated cases [2].

About 10% of AP cases are linked to infectious agents, most commonly viral pathogens; other causes - including anatomic, obstructive (including biliary), trauma, toxins, metabolic, systemic illnesses, inborn errors of metabolism, genetic predispositions, and idiopathic - have all been described as potential etiologies in pediatric AP, acute recurrent pancreatitis (ARP), and chronic pancreatitis (CP) [2,5]. Determining the exact cause after diagnosis is essential, as it guides the choice of treatment and targeted interventions, which in turn influence the overall clinical outcome [5]. Because the measles, mumps, and rubella (MMR) vaccine is a live attenuated vaccine, pancreatitis is listed among gastrointestinal adverse events reported in post‑approval surveillance for M-M-R II (Measles, Mumps, and Rubella Virus Vaccine Live) [6]. However, it is not emphasized in routine patient‑facing vaccine information, which suggests that such events are extremely rare [7,8].

Here, we describe a case of a 12-month-old girl who developed AP three days after receiving the MMR vaccine, and we summarize prior published reports to contextualize the rarity of this temporal association.

## Case presentation

Patient information

A 12-month-old female child, previously healthy, with no prior hospitalizations, no chronic medications, and no known allergies, presented to the emergency department with acute vomiting and vigorous crying. Birth history revealed that she was a term infant, delivered by cesarean section, with a birth weight of 3 kg and no neonatal intensive care unit (NICU) admission. Vaccinations were up to date as per the caregiver's report, including receipt of the MMR vaccine three days prior to presentation.

She presented with sudden, intense, vigorous crying (interpreted as abdominal pain/discomfort), followed by five episodes of non-projectile vomiting, with symptom onset occurring earlier on the same day of presentation. Vomitus consisted of milk contents, with no blood and no bilious (green) content. The first three episodes were of larger volume, followed by two smaller episodes. Her appetite decreased afterward.

The caregiver reported difficulty breathing beginning with symptom onset (no cyanosis or desaturation documented; peripheral oxygen saturation (SpO_2_) remained normal in the emergency department). There was no fever, diarrhea or constipation, jaundice, change in bowel habit, or urinary symptoms. There were no upper respiratory tract infection (URI) symptoms, rash, trauma/falls, recent travel, history of toxin or medication ingestion, or sick contacts. She had no prior similar episodes and no family history of pancreatitis, hyperlipidemia, or autoimmune disease.

Examination

On arrival, vital signs were stable: temperature was 36.2°C, heart rate (HR) was 125/min, respiratory rate (RR) was 38/min, SpO_2_ was 100% on room air. She was alert, well perfused (capillary refill time (CRT) <2 seconds), and not toxic.

Abdominal findings evolved with time. In the emergency department, she was noted to have epigastric tenderness despite settling after ondansetron administration. Later inpatient documentation described a soft, non-distended abdomen with normal bowel sounds and no focal tenderness, consistent with improving symptoms.

Weight, length, and head circumference follow expected growth curves. No dysmorphic features were noted. Head and neck examination revealed a normocephalic head, and the ears, nose, and throat appeared normal. The chest was clear with normal breath sounds, and the cardiovascular examination revealed normal heart sounds without murmurs. Genitalia were appropriate for age and sex. Neurologically, the child had normal tone and reflexes.

Investigations

Capillary blood gas showed mild metabolic acidosis (pH 7.31, bicarbonate (HCO_3_^-^) 18 mmol/L) and lactate 3.2 mmol/L, suggesting physiologic stress/dehydration risk in the context of vomiting. Abdominal X-ray was unremarkable.

**Figure 1:**
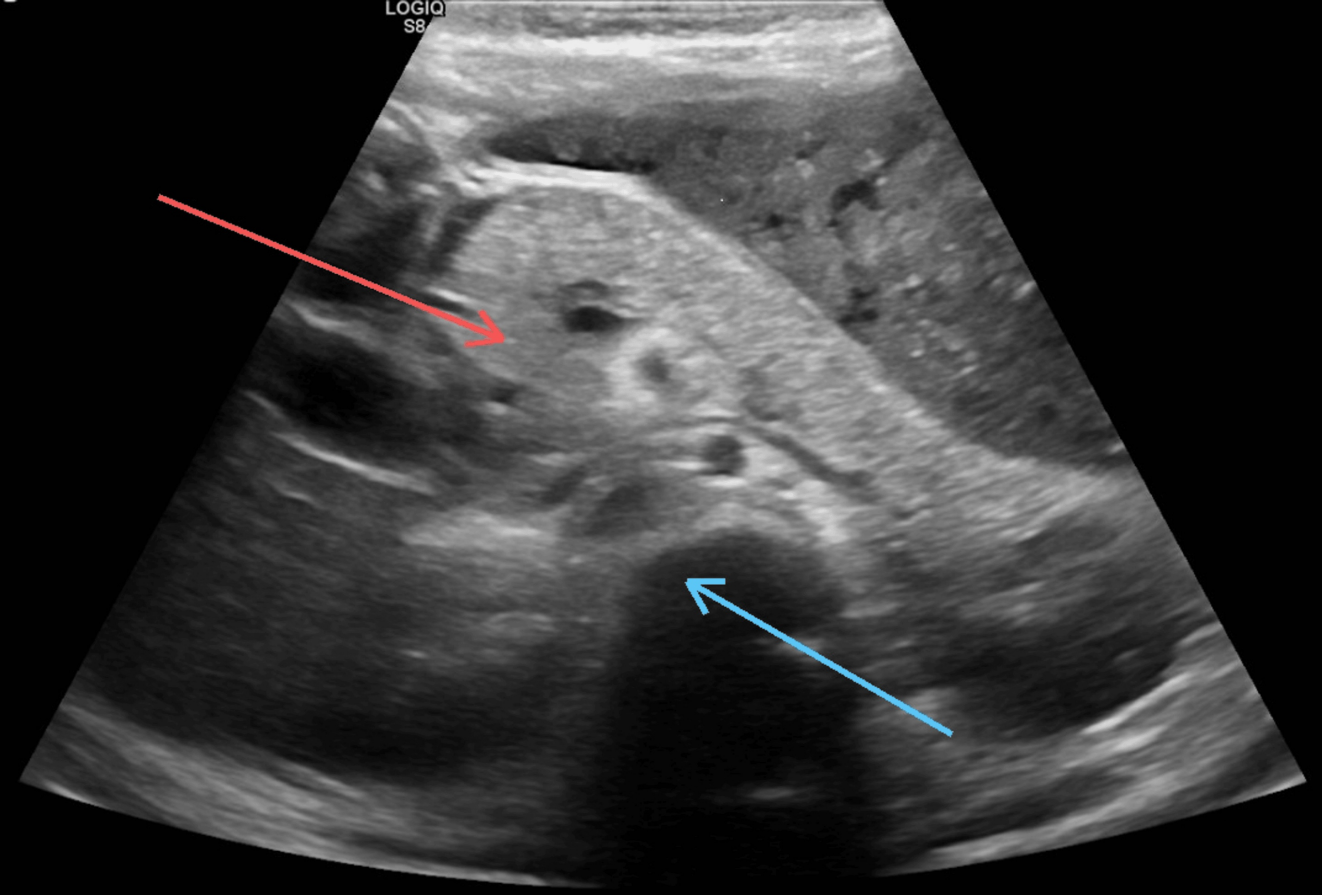
Ultrasound of the abdomen The red arrow indicates a bulky pancreas. The blue arrow indicates peripancreatic fluid.

Ultrasound of the abdomen on admission demonstrated a bulky pancreas with peripancreatic fluid and minimal free peritoneal fluid, findings consistent with AP. It also reported prominent common bile duct (CBD) and gallbladder sludge; radiology discussion documented no obstruction, and CBD prominence was considered inflammatory (Figure 1). Serum pancreatic enzymes were markedly elevated (lipase 2451 IU/L, amylase 331 IU/L) and trended down by discharge (lipase 1692 IU/L, amylase 316 IU/L) (Table 1). 

**Table 1:** Key investigations during admission *Laboratory tests done on day 3. PT: prothrombin time; INR: international normalized ratio; pCO_2_: partial pressure of carbon dioxide; HCO_3_^-^: bicarbonate; WBC: white blood cells; CRP: C‑reactive protein; ALT: alanine aminotransferase; AST: aspartate aminotransferase; ALP: alkaline phosphatase; GGT: gamma-glutamyl transferase

Laboratory test	Day 1	Day 2/3	Reference range
WBC	11690 /µL	-	6000-17500 /µL
CRP	2.8 mg/L	-	0-10 mg/L
Sodium	139 mmol/L	141 mmol/L	135-145 mmol/L
Potassium	4.1 mmol/L	4.3 mmol/L	3.5-5.5 mmol/L
Calcium	2.32 mmol/L	2.27 mmol/L	2.12-2.52 mmol/L
Lipase	2451 IU/L	1692 IU/L*	0-160 IU/L
Amylase	331 IU/L	316 IU/L*	25-125 IU/L
ALT	142 IU/L	87 IU/L	7-45 IU/L
AST	181 IU/L	90 IU/L	15-37 IU/L
ALP	298 IU/L	264 IU/L	142-335 IU/L
GGT	-	80 IU/L	5-31 IU/L
Albumin	38.64 g/L	36.01 g/L	35-52 g/L
PT	10.6 s	11.2 s	9-13 s
INR	0.85	0.90	0.8-1.2
pH (capillary blood gas)	7.31	-	7.35-7.45
pCO_2_ (capillary blood gas)	36 mmHg	-	35-45 mmHg
HCO_3_^- ^(capillary blood gas)	18 mmol/L	-	22-26 mmol/L
Lactate (capillary blood gas)	3.2 mmol/L	-	<2.0 mmol/L
Cytomegalovirus IgM	Non-reactive	-	Non-reactive
Epstein-Barr virus IgG	Negative	-	Negative
Epstein-Barr virus IgM	Negative	-	Negative

Management and hospital course

She met the INSPPIRE diagnostic criteria for AP (≥2 of three criteria): pain equivalent symptoms/tenderness, lipase ≥3× ULN, and supportive ultrasound findings [1]. Standard evaluation for common etiologies of AP ruled out anatomical, traumatic, metabolic, and toxic causes. Regarding infectious causes, cytomegalovirus (CMV) and Epstein-Barr virus (EBV) serology were obtained and were non-reactive. However, mumps-specific investigations (polymerase chain reaction (PCR) or acute/convalescent IgM/IgG serology) and broader viral screening (coxsackievirus, adenovirus, parvovirus B19) were not performed. As no alternative identifiable etiology was found on initial workup and given the close temporal relationship to MMR vaccination, a vaccine-associated etiology was considered the most likely explanation.

She was admitted to the pediatric ward for supportive management, including intravenous (IV) fluids (~1.5× maintenance), initial nil per os (NPO), antiemetics, and close monitoring (vitals, oxygenation, hydration status, and strict input/output). Surgical causes of the acute abdomen were ruled out. She improved over three days of hospital observation and soon began tolerating oral intake. Enzyme levels trended down. Gallbladder sludge was addressed as a possible associated finding (ursodeoxycholic acid was prescribed for a short course), but imaging discussion did not support obstructive biliary disease as the primary driver in this admission.

Over the year following discharge, she remained well on multiple telephone follow-ups, tolerated feeds normally, and experienced no further abdominal pain or vomiting.

## Discussion

Published evidence linking pancreatitis to the MMR vaccine is largely limited to isolated case reports, underscoring how uncommon this temporal association appears to be in the medical literature. 

One of the earliest reports is by Adler et al. (1991) describing pancreatitis occurring after MMR vaccination in an adult patient [9]. Furthermore, Toovey and Jamieson (2003) reported pancreatitis following combined MMR immunization. They discussed the sparse literature available at the time, emphasizing that only a small number of similar cases had been reported [10]. In pediatrics, Hansen et al. (2003) described a 12-year-old who developed AP after a second MMR dose, with symptom onset reported at approximately three weeks post-vaccination and imaging features including pancreatic edema and biliary duct changes without a clear alternative cause after evaluation [11]. 

A related vaccine-associated pancreatitis is described by Feldman and Zer (2000), who reported a 13-month-old child with AP after mumps vaccination, presenting like an acute surgical abdomen, again framed as an extremely rare event [12]. More recently, Verma et al. (2017) reported a 15-month-old who developed AP after MMR vaccination and subsequently formed a pancreatic pseudocyst. Importantly, the authors explicitly highlighted the scarcity of published cases and noted that only a very limited number of prior reports of pancreatitis after MMR were identifiable in the literature at that time, supporting the conclusion that this presentation is rare [13].

Even with a tight temporal relationship, pediatric guidance encourages evaluation for common causes, including biliary disease, medications/toxins, trauma, anatomic abnormalities, metabolic triggers (hypertriglyceridemia/hypercalcemia), and infection [2]. 

However, in this case, the infant's clinical course (rapid improvement, no organ dysfunction, down-trending enzymes) was consistent with mild AP, and supportive inpatient care with monitoring and refeeding once tolerated aligned with North American Society for Pediatric Gastroenterology, Hepatology, and Nutrition (NASPGHAN) recommendations [2,3]. Moreover, ultrasound noted gallbladder sludge and a prominent CBD, not suggestive of obstructive biliary pancreatitis; although sludge can be clinically relevant and warrants follow-up imaging and laboratory review.

To formally assess the likelihood of a causal relationship, we used the Naranjo Adverse Drug Reaction Probability Scale [14]. This case scored 3, placing the association in the "possible" category (score 1-4). Criterion 2 was met (+2) given the temporal relationship between MMR administration and symptom onset. Prior published case reports of MMR-associated pancreatitis fulfilled criterion 1 (+1) [9-13]. Criteria for de-challenge (criterion 3) and objective confirmatory evidence (criterion 10) were scored 0: spontaneous resolution of mild AP is the expected clinical course regardless of etiology and cannot be attributed to vaccine "withdrawal," and mumps-specific investigations (PCR or acute/convalescent serology) were not obtained during the acute episode, precluding objective confirmation of vaccine-strain involvement. Re-challenge (criterion 4) was ethically unjustifiable and was not performed. Similarly, under the World Health Organization-Uppsala Monitoring Centre (WHO-UMC) causality classification, this case would be categorized as "possible" - a plausible time relationship exists, an alternative cause cannot be confirmed but also cannot be excluded, and more specific information for a fuller assessment was unavailable [15].

It is equally important to acknowledge that idiopathic pancreatitis constitutes a recognized and not insignificant proportion of AP cases in the pediatric population, with some series reporting rates of 15-30% in children where no clear etiology is identified despite thorough evaluation [16]. In infants specifically, atypical presentations and limited investigative yield may further inflate this proportion. This background rate of unexplained pancreatitis means that temporal associations with preceding events, including vaccinations, may be coincidental and reinforces why causality cannot be assumed from timing alone.

Furthermore, AP in infants is an uncommon and often diagnostically challenging condition due to its atypical presentation. This case highlights the importance of systematic evaluation, and such rare presentations should still be acknowledged and considered in the differential diagnosis. Such presentations also raise questions regarding subsequent vaccinations of the child, particularly the second dose of MMR. Given the scarcity of available data, it remains difficult to determine whether future vaccinations should be administered in such infants.

## Conclusions

AP in infants is a rare and often difficult diagnosis, particularly due to its nonspecific clinical presentation. While most cases have identifiable and more common etiologies, this report highlights that uncommon associations may occasionally be encountered. A temporal association was observed, but causality could not be established; such reports could be coincidental given the background incidence of idiopathic pancreatitis. However, recognizing these rare possibilities supports a more comprehensive and thoughtful clinical approach, while maintaining adherence to established guidelines and reinforcing confidence in the overall safety of routine immunizations.
